# Placental Transcriptome Analysis in Connection with Low Litter Birth Weight Phenotype (LBWP) Sows

**DOI:** 10.3390/genes15060703

**Published:** 2024-05-28

**Authors:** Julia Linck Moroni, Stephen Tsoi, Irene I. Wenger, Graham S. Plastow, Michael K. Dyck

**Affiliations:** Department of Agricultural, Food & Nutritional Science, University of Alberta, Edmonton, AB T6G 2P5, Canada; linckmor@ualberta.ca (J.L.M.); scmtsoi@gmail.com (S.T.); iwenger@ualberta.ca (I.I.W.); plastow@ualberta.ca (G.S.P.)

**Keywords:** low litter birth weight phenotype, mRNA-Seq, porcine, placenta, transcriptome, transcriptional factor binding site

## Abstract

It is possible to identify sub-populations of sows in every pig herd that consistently give birth to low birth weight (BW) piglets, irrespective of the litter size. A previous study from our group demonstrated that placental development is a main factor affecting the litter birth weight phenotype (LBWP) in sows, thereby impacting the BW of entire litters, but the biological and molecular pathways behind this phenomenon are largely unknown. The aim of this study was to investigate the differential gene expression in placental tissues at day 30 of gestation between low LBWP (LLBWP) vs. high LBWP (HLBWP) sows from a purebred Large White maternal line. Using mRNA sequencing, we found 45 differentially expressed genes (DEGs) in placental tissues of LLBWP and HLBWP sows. Furthermore, (GO) enrichment of upregulated DEGs predicted that there were two biological processes significantly related to cornification and regulation of cell population proliferation. To better understand the molecular interaction between cell proliferation and cornification, we conducted transcriptional factor binding site (TFBS) prediction analysis. The results indicated that a highly significant TFBS was located at the 5′ upstream of all four upregulated genes (*CDSN*, *DSG3*, *KLK14*, *KRT17*), recognized by transcription factors EGR4 and FOSL1. Our findings provide novel insight into how transcriptional regulation of two different biological processes interact in placental tissues of LLBWP sows.

## 1. Introduction

The low litter birth weight phenotype (LLBWP) is a sow-related phenotype expressed in piglets, in which sows consistently produce low birth weight (BW) litters across parities [1]. In contrast to individual piglet low BW, LLBWP affects the growth performance of entire litters. Recent research has demonstrated that litter average BW shows between 40% and 60% repeatability, with some sows consistently producing low BW piglets over multiple parities [2,3,4]. Nonetheless, the phenotype is passed down through generations, perpetuating the problem in pig production systems [4].

The BW phenotypic outcome of the litter is dependent on adequate placental morphogenesis and intrauterine environment [5,6]. The conceptuses are dependent upon the placenta for the regulation of nutrients, gases, and waste exchanges between them and the maternal system. In turn, the functional capacity of the placenta to supply these demands is under the control of both the fetal and maternal genomes [7]. Furthermore, establishment of pregnancy requires a close physical and molecular communication between the conceptuses and the maternal reproductive tract that begins during implantation and continues until the placenta is fully formed [8]. In a recent study from our lab, physiological data showed that the uterine capacity in LLBWP sows was below the threshold for optimal placental development, which in turn affected the placental volume and embryonic development [9]. However, there is clearly more to placental development than changes in placental volume during gestation. With that in mind, the ability to identify candidate genes and biological pathways, together with the physiological data previously analyzed, is expected to facilitate a better understanding of how the litter birth weight phenotype (LBWP) affects these traits. 

In the past, genotyping to detect single nucleotide polymorphisms (SNPs) has been the most common approach to finding genetic variation for specific traits in porcine reproduction. Two different strategies are used to detect the effect of specific genes on a trait, such as BW [10,11]. The first uses linkage analyses to detect regions harboring the genes related to the trait [12]. The second identifies candidate genes via their physiological role in reproduction or locates the genomic region linked with a phenotype [13]. Significant association has been shown for litter BW and individual BW with regions on *Sus scrofa* (SSC) chromosomes 1, 4, 5, 6, and 7 [14]. Zhang et al. (2018) [4] analyzed the genotypes of sows from the same population used in this study and found associations with both individual and litter BW on SSC1, SSC9, and SSC19. 

Although these genetic linkage studies improve the understanding of genomic mutation linked with a certain phenotype, the molecular mechanisms determining the LBWP in sows during placental development are still unclear. The purpose of this study was to obtain a representative placental transcriptomic profile of LLBWP sows at day 30 of gestation. We focused particularly on the differential gene expression in placental tissues comparing sows with a LLBWP vs. a high litter birth weight phenotype (HLBWP) using mRNA-seq. More specifically, we selected significant DEGs in LLBWP sows to identify gene markers and uncover potential molecular mechanisms that may be related to the insufficient placental function that appeared in LLBWP sows.

## 2. Materials and Methods

### 2.1. Sow Selection and Tissue Collection

All animal procedures were conducted at the University of Alberta Swine Research and Technology Centre (SRTC, Edmonton, AB, Canada) with the approval of the Faculty Animal Policy and Welfare Committee–Livestock (Protocol—AUP00002650). The selection of sows and collection of tissues for this study has been previously described [9]. Briefly, 40 purebred Large White sows (*n* = 20 LLBWP and *n* = 20 HLBWP) from a nucleus pig breeding facility (Hendrix Genetics, Tullymet Nucleus Unit, Balcarres, SK, Canada) were selected based on their reproductive and piglet BW history and their expression of a clear LLBWP and HLBWP over at least two successive parities. The sow selection concentrated on the bottom 12% and top 12% of the population, from which the average litter BW was 1.20 kg for LLBWP and 1.50 kg for HLBWP. The sows were received at the SRTC facility and were bred with semen from purebred Large White boars of proven fertility (Hendrix Genetics) on their second estrus following altrenogest withdrawal (Matrix^TM^, Merck AH, Kenilworth, NJ, USA) to synchronize estrous cycles. Sows were euthanized on day 28 to 30 of gestation (mean ± s.d; day 29.15 ± 0.6) and individual samples of placenta and embryos were collected. Total number of embryos (TNE), embryo weight (EW), embryonic viability, and crown–rump length (CRL) measurements were recorded, along with the ovulation rate (OR) and allantochorionic fluid volume (AFV), which were determined as described in our previous study [9]. Tissues were frozen in liquid nitrogen and stored at −80 °C for later analysis.

### 2.2. RNA Preparation

Placental tissues were processed into powder as previously described [15]. A small amount (0.05 mg) of powdered placental tissue was transferred into a 2 mL micro-centrifuge tube containing 500 μL of Nucleozol^®^ Reagent (MACHEREY-NAGEL, Bethlehem, PA, USA) and homogenized into lysates. Total RNA was purified from lysates using NucleoSpin^®^ RNA silica spin columns (MACHEREY-NAGEL, Bethlehem, PA, USA) as per the manufacturer’s instructions. Total RNA QC was performed using Agilent 2200 TapeStation System (Agilent, Santa Clara, CA, USA) and NanoDrop (Thermo, Pleasanton, CA, USA). At least 1 μg of high-quality total RNA (RNA integrity number (RIN) > 8.0) from each sample was purified for mRNA isolation, using poly-T-oligo attached magnetic beads. RNA sequencing (RNAseq) including library construction and bioinformatics analysis was performed at BGI (Shenzhen, China). RNA quality for five samples did not meet minimum quality requirements and these were not submitted for RNA seq (*n* = 35; 18 LLBWP and 17 HLBWP).

### 2.3. Library Template Construction and Sequencing

The mRNA was cleaved into small fragments using divalent cations under elevated temperature. Double-stranded cDNA was created using random priming with reverse transcriptase, DNA polymerase I and RNase H (as per DNBSeq™ protocols). After adaptor ligation to both ends of cDNA molecules, cDNA enrichment was carried out by PCR. The double-stranded PCR products were heat denatured and circularized by the splint oligo to create a final library of single-strand DNA circles (ssDNA circle). Finally, these ssDNA circle molecules were used to create DNA nanoballs (DNBs) by rolling circle replication (RCR) and loaded into the patterned nanoarrays with pair-end reads of 100 bp to read through on the BGISEQ-500 platform (BGI, Shenzhen, China). DNBSeq™ is a recent next-generation sequencing platform and the library template construction used DNB and combinatorial probe–anchor synthesis (cPAS), giving a higher signal-to-noise ratio, higher spot densities, and faster sequencing times than alternative methods [16].

### 2.4. Sequence Mapping and Gene Quantification

For this project, the BGISEQ-500 next-generation sequencing platform (Beijing Genomics Institute, Shenzhen, China) was used to generate raw sequence reads. After using SOAPnuke software (v1.5.2, https://github.com/BGI-flexlab/SOAPnuke, accessed on 16 September 2019), reads from adaptors, unknown bases (N) and low-quality sequences (low-quality read defined as the percentage of base in which quality is less than 20% and greater than 40% in a read) were removed. Finally, clean reads were stored in FASTQ format. HISAT (v2.0.4) [17] was used to map clean reads to the reference genome (Sscrofa 11.1) and Bowtie2 (v2.2.5) [18] was used to map clean reads to reference transcripts. Gene expression levels were calculated using the transcripts per million (TPM) method provided by RSEM software (v1.2.12) [19]. All sequences data were deposited to the NCBI SRA (Sequence Read Archive) database at BioProject with accession number: PRJNA608736. 

### 2.5. Differentially Expressed Gene Detection

Differentially expressed gene (DEG) detection was performed by the comparison of transcripts between LLBWP and HLBWP sow groups using the DEseq2 algorithm [20] for measuring the gene expression levels via TPM values and filtering DEGs. The criteria for filtering DEGs were as follows: Log2 fold change (FC) ≥ 1 (upregulated genes) or ≤1 (downregulated genes) and adjusted *p*-value ≤ 0.05. 

### 2.6. Functional Enrichment Analysis for DEG and TFBS Prediction

Using gene ID from the DEGs of day 30 (D30) placental tissues to identify gene symbol (GS) and GenBank accession numbers (GA#) for pigs, the correct identification of human GS and the same GS in pigs was performed by Blast (v2.10.0) search. Enriched gene ontology (GO) terms analysis of all DEGs was conducted using GOnet (https://tools.dice-database.org/GOnet/, accessed on 6 January 2020) [21]. Fisher’s exact test was applied to identify the significant GO categories or biological processes, selecting the analysis type for GO term enrichment. Corrected *p*-values calculated according to FDR control procedure were considered significant when lower than 0.05, calculated according to FDR control procedure [22]. The 1 KB upstream sequences of the 5′ non-coding region of *CDSN*, *DSG3*, *KLK14*, and *KRT17* were obtained from NCBI for TFBS prediction using AnimalTFDB3.0 [23].

### 2.7. Quantitative Real-Time RT-PCR Validation

A two-step quantitative real-time RT-PCR (RT-qPCR) was performed for validation purposes. Six RNA samples from placental tissues (LLBWP, *n* = 3 and HLBWP, *n* = 3) were selected using the same samples as for RNA-seq. The first strand of cDNA synthesis started with 500 ng of total RNA after ezDNase treatment to remove DNA before using it in 20 μL of the SuperScript™ IV VILO™ Master Mix (Invitrogen, Waltham, MA, USA). After synthesis, 2 μL of the total cDNA reaction mixture was taken as a template to perform the real-time qPCR with PrimeTime^®^ RT-PCR Kit (IDT, Coralville, IA, USA) in 10 μL total volume of qPCR according to the instruction manual for the QuantStudio Flex 6 system (Thermo Fisher Scientific, Pleasanton, CA, USA). All samples were assayed in duplicate wells. The Fast program was used as follows: 95 °C for 3 min, followed by 45 cycles of 95 °C for 5 s, and 60 °C for 30 s. Cycle threshold values and primer efficiencies were obtained from SDS2.3 Software (Applied Biosystems, Pleasanton, CA, USA) installed in the system by performing auto-setting for threshold cycle (Ct) and baseline calculation. 

Three DE genes (*CDSN*, *HBEGF*, *PDPN* from placenta tissues) were validated by RT-qPCR. The PrimerQuest tool from IDT (https://www.idtdna.com/Primerquest/Home/Index, accessed on 16 March 2020) was used to design primers and probes for both DE and reference genes (*HPRT1*, *PGF*). Validation of the final ideal primer and probe sequences was conducted to ensure they were not located within the same exon using Primer-Blast from NCBI (Primer-Blast frg 24, 2015) [24]. The sequence information for the primers can be found in Supplementary Table S1. For RT-qPCR analysis, the Relative Expression Software Tool 2009 (REST; http://rest.gene-quantification.info/, accessed on 13 April 2020) was used to implement a randomized test [25] and to assess statistical significance of the up- or downregulation of the target genes after normalization to the reference gene. Statistical analyses were considered significant when *p* ≤ 0.05.

## 3. Results

### 3.1. Sequencing Quality, Transcript Mapping, and Annotation

**Figure 1:**
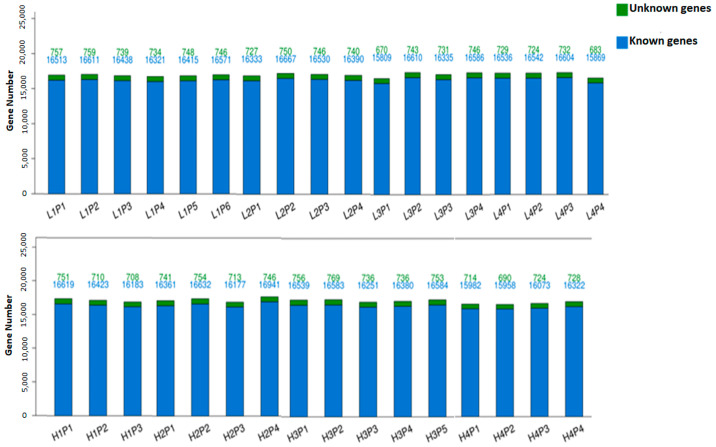
Uniformity of gene mapping. *Y*-axis and *X*-axis represent gene number and individual sample names, respectively. Each bar column represents a sample with color legend indicating the number of unknown and known genes listed at the top, with green indicating unknown and blue indicating known genes. Expression threshold of detected genes was established as an expected count of >1.

Sequencing reads generated by the BGISEQ 500 platform were assessed for quality and reads with low quality, including adaptor sequences and unknown base reads, were removed before mapping to the porcine reference genome. Clean read quality metrics of all placental samples (*n* = 35; LLBWP = 18 and HLBWP = 17) are shown in Supplementary Table S2. On average, 8.2 Gb clean bases with Q20 and Q30 greater than 90% and 80% were obtained, respectively. After mapping clean reads to the pig reference genome and transcripts, total mapped reads and total gene and transcript number were assessed (Supplementary Table S3). The uniformity of the mapping result for each sample is shown in Figure 1. On average, 17,140 genes were identified, of which 16,407 were known genes and 733 were unknown genes. Full annotation of all 35 samples including level of gene expression in TPM, gene ID, transcript ID, length, expected counts, gene symbol, and description are shown in Supplementary Table S4. 

### 3.2. DEG Analyses in Placental Tissues

**Figure 2:**
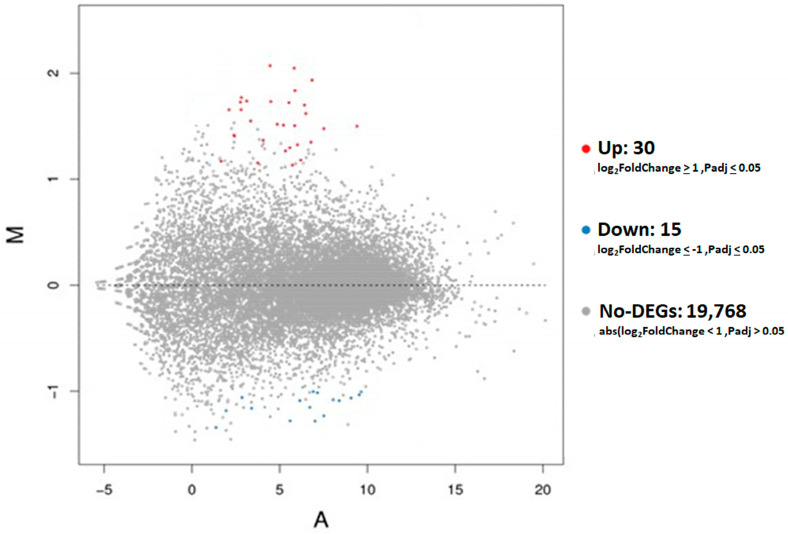
MA plot of HLBWP vs. LLBWP in placental tissues. Significant DEGs generated by DESeq2 analysis with red dots represent significantly upregulated DEGs and blue dots indicate downregulated genes in the placental tissues.

The DEseq2 algorithm was used to identify DEG of D30 placental tissues between LLBWP and HLBWP. An MA plot was generated to visualize the quantitative differences in gene expression comparing LLBWP to HLBWP (Figure 2), showing a total of 45 DEGs in placental tissues (Supplementary Table S5) with 15 downregulated genes (green dots) and 30 upregulated genes (red dots) in LLBWP compared to HLBWP. 

### 3.3. Functional Enrichment Analysis of DEGs in Placental Tissues

**Figure 3:**
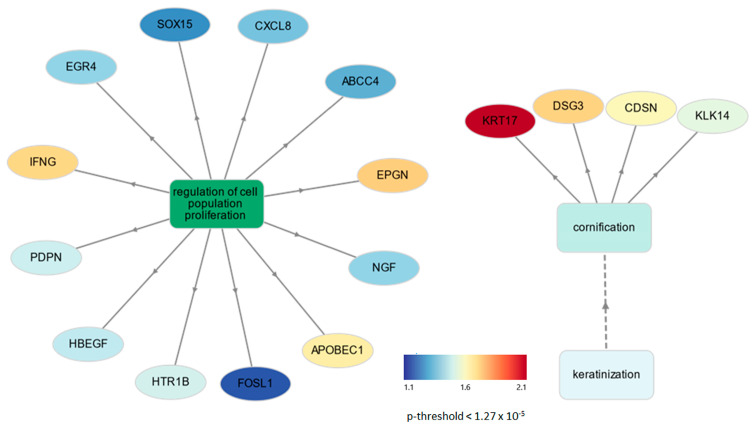
Network connection among upregulated DEGs interacting with each other from different biological processes in placental tissues. *p*-value threshold value was considered significant when lower than 0.05 according to Fisher’s exact test from GOnet. There were two apparently distinguishable types of nodes: GO terms (rectangle) and genes (circular). Arrows point from GO term towards the gene. Dotted arrow indicates the less specific term to the more specific term.

GO term enrichment using GOnet was performed on upregulated DEGs in placental tissues selected with a background gene list as the porcine reference gene list (Supplementary Table S6) at q-value threshold ≤ 0.05. Two biological processes related to cornification (with *CDSN*, *DSG3*, *KLK14*, and *KRT17*) and regulation of cell population proliferation (Supplementary Table S7) were found in placental tissues. No statistically significant cellular components and molecular functions were found. A graphical network output shows how upregulated DEGs interact with each other from different biological processes in placental tissues (Figure 3) comparing LLBWP to HLBWP. 

### 3.4. Transcriptional Factor Binding Site Prediction for Four Genes Involved in Cornification

The non-coding region of *CDSN*, *DSG3*, *KLK14*, and *KRT17* was used to identify potential binding transcription factors for nucleotide sequences at the 5′ end of these genes. The results showed that two transcriptional factors, EGR4 and FOSL1 (Supplementary Table S8), were identified to recognize transcriptional binding motifs at the 5′ end of these genes. 

### 3.5. Validation of DEG Data by RT-qPCR

The LBWP (LLBWP and HLBWP) effect on the expression of some genes related to the main GO terms from placental tissues was confirmed by RT-qPCR (Table 1). The expression of *CDSN*, *HBEGF*, and *PDPN* confirmed their upregulation in placental tissues in LLBWP when compared to HLBWP.

## 4. Discussion

Sows displaying the LLBWP tend to produce litters with generalized low BW [5]. In the present population of sows, for example, 50% of the litters produced by LLBWP sows had an average individual BW of <1.2 kg, and there were no litters with BW ≥ 1.5 kg [9]. Therefore, the group of sows displaying this phenotype is believed to make the most substantial contribution to the low BW of piglets and the variation in postnatal growth performance, independent of the total number of piglets born in a litter [5]. Knowing that this trait is repeatable over parities and passed to the next generation of replacement gilts in the breeding herd, the ability to understand the biological and molecular mechanisms that are driving this scenario can be directed toward selection to increase the efficiency of the pig breeding herd [2,3,4,5]. As described in our previous study, the physiological data showed that the uterine capacity in LLBWP is below the threshold for optimal placental development, which in turn affects placental volume and embryonic development [9]. Therefore, the main goal of the study was to understand the molecular mechanisms and biological pathways that are driving the LLBWP at the placental level. The ability to understand normal maternal–embryonic dialogue at the molecular level in the placental tissues is critical to developing breeding strategies that improve embryonic and fetal development. 

### 4.1. Genes Differentially Expressed in Placental Tissues from LLBWP Sows

The placental interface mediates the interaction between the mother and the conceptuses. Gene expression studies have been performed in several species to examine how changes in gene expression participate in the crosstalk between the maternal and embryonic tissues [26]. Placental efficiency is known to be regulated by a variety of factors, including the surface area of exchange, the thickness of the exchange barrier, blood flow at both the maternal and conceptuses sides and the number and efficiency of transporters [7]. According to our GO term enrichment result, two biological processes, cornification (*CDSN*, *DSG3*, *KLK14*, *KRT17*) and regulation of cell proliferation (*ABCC4*, *APOBEC1*, *CXCL8*, *EGR4*, *EPGN*, *FOSL1*, *HBEGF*, *HTR1B*, *IFNG*, *NGF*, *PDPN*, *SOX15*) were involved in the LLBWP sows’ placental function, and these DEGs were all upregulated. Some of the upregulated genes also seemed to be involved in tissue morphogenesis, angiogenesis, and nutrient transport activity and the immune function. Any unbalanced mechanism by which each one of the genes acts in these processes, however, may appear to have negatively affected the placental function in the LLBWP sows.

### 4.2. Impact of Placental Tissue Cornification and Cell Proliferation in LLBWP Sows

In pigs, throughout gestation, the fetuses’ increased demand for nutrients is met by the remodeling of placental folds and increased blood flow [6,27]. Angiogenesis occurs mainly during two waves; the first one is during the post-implantation period to day 20 of gestation, and the second from days 50 to 70 [28]. As a result, proper regulation of placental angiogenesis seems to influence the efficiency through which the establishment and maintenance of pregnancy occurs [29,30]. The process is regulated by a complex range of genes that have the ability to improve placental angiogenesis and consequently support placental development at critical stages of gestation. According to our findings, placenta from LLBWP sows expressed a group of genes that appear to be related to deficient angiogenesis processes and nutrient support. 

Among them, the product of the *PDPN* gene, podoplanin (PDPN), is recognized to be part of the regulation of cell proliferation and wound healing [31]. This gene affects the mucin-type transmembrane protein, and in humans, it is known to play an essential role in a variety of physiological and pathological processes such as angiogenesis, inflammation, thrombus formation, and cancer progression, as well as in cellular adhesion, migration and chemotaxis [31,32]. PDPN is believed to be related to fetal vessel angiogenesis during the placental development. Altered expression of this gene may be related to impaired fetal interstitial fluid homeostasis and impaired angiogenesis [33]. Nonetheless, *PDPN* expression was found to be upregulated during ischemia–hypoxia, inflammation, and in cases of pre-eclampsia in humans [32]. 

Another pathway that directly influences adequate blood flow and nutrient transport is the cornification of placentas from LLBWP. Cornification is a natural process that drives toward programmed cell death, which results in corneocytes and lipids, essential to the cornified skin layer’s resistance, elasticity, and water repellence [34]. However, in the case of placental tissue, if exacerbated, this process can create a barrier and interfere in the crossing of nutrients and gases [35]. Among our findings, *CDSN* (*corneodesmosin*) expression was upregulated in LLBWP placental tissues. Garrido-Gomez et al. (2017) [36] found an upregulation of this gene in human chorion with reduced blood perfusion. The reduced uterine perfusion is explained by the fact that the CDSN molecule is the major component related to the cornification of epithelial layers. In our research, another three genes associated with cornification were upregulated in LLBWP sows, *KRT17* (*keratin 17*) and *KLK14* (*kallikrein-related peptidase 14*) and *DSG3* (*desmoglein 3*). *DSG3* is also known to be related to cellular apoptosis, programmed cell death, cleavage of cellular proteins, cornification, and keratinization [37]. *KLK14* is a member of the kallikrein subfamily of serine proteases that have diverse physiological functions such as regulation of blood pressure, desquamation, and immune response [38,39]. A previous study has observed *KLK14* expression localized in porcine uterine luminal and glandular epithelium, and stroma throughout the endometrium after day 10 of development [40]. Whether upregulated *KLK14* in D30 placental tissues of LLBWP reflects the homeothesis balance of keratinization in pigs through desquamation is not known. Recent studies indicated that keratin 17 overexpressed in keratinocytes not only causes psoriasis but also promotes epithelial proliferation and tumor growth [41]. We propose KRT17 may cause a similar mechanism in porcine placental tissues, promoting cell proliferation and inflammation.

The primary function of chemokines is to command immune cell migration into infected or inflamed tissue to initiate an effective immune response [42]. They also play a role in angiogenesis and hematopoiesis, and regulate activation, proliferation, differentiation, and apoptosis in the cells they attract [43,44]. Functional chemokines and their receptors are widely expressed in maternal–conceptus tissues and are a major player in tissue communication and pregnancy success [45]. Reproductive success relies on the ability of the maternal tract to remain tolerant to the fetuses and at the same time to protect them from infections [46]. To achieve this goal, appropriate communication has to be established and maintained. The adverse effects of pro-inflammatory cytokines during human and mouse pregnancy are already well known [47,48,49]. In women, these pathways can lead to endothelial cell injury, reduced blood supply, and subsequent embryonic death, in addition to deficient angiogenesis [50,51]. The latter studies revealed that an elevated concentration of pro-inflammatory cytokines attack maternal endothelial cells, ultimately restricting blood supply to an already stressed conceptus. 

Within our findings, the chemokine-mediated signaling pathway and immune system processes in LLBWP are hypothesized to be working together. *CXCL8* (*C-X-C motif chemokine ligand 8*) was found to be upregulated in LLBWP. Also known as *IL8* (*interleukin 8*), it is a member of the chemokine family, and it is a major mediator of inflammatory response. The encoded protein is secreted by neutrophils, where it serves as a chemotactic factor by conducting the neutrophils to the site of infection [52]. CXCL8 was found to be elevated during pre-eclampsia in women and pre-term labor as a result of an overall pro-inflammatory reproductive environment [53,54]. Another finding was related to the upregulation of *IFNG* (interferon-γ), which is a proinflammatory cytokine secreted in the uterus during early pregnancy, and it is produced by uterine natural killer cells in the maternal endometrium [55]. Porcine embryonic and fetal loss has been associated with an elevation of *IFGN* expression [28] in a study that found highly elevated expression of *IFGN* in biopsies of days 15 to 23 of gestation on attachment sites of viable retarded conceptuses compared to healthy littermate sites. According to the authors, IFNG, through immune-mediated mechanisms, may compromise the first wave of angiogenesis immediately after the implantation period, causing conceptus stress and subsequent growth retardation and loss. In addition, *NGF* (*nerve growth factor*), which is a neurotrophin associated with diseases of the immune system and inflammation, was also upregulated in LLBWP. Jana et al. (2012) [56] found the expression of *NGF* significantly increased in gilts with induced endometritis compared to sows with a normal uterine environment. Altogether, based on these studies, chemokines and immune responses at the maternal–embryonic interface may be overactive in LLBWP sows and, instead of protecting the embryos, this creates an unsuitable environment for adequate embryonic development. 

### 4.3. Placental Compensation Mechanisms in LLBWP

Researchers have shown that placentas linked to low weight fetuses tend to exert compensatory mechanisms, mainly during the second third of the gestational period, to overcome the growth development hurdle [29,30,57]. In pigs, even though it is not invasive, the interdigitation of the trophectoderm into the endometrium is progressive throughout gestation and tends to increase until the placenta is entirely covered [58]. Following this finding, Vallet and Freking (2007) [57] mentioned that as a compensatory mechanism to increase total surface area, lighter fetuses showed deeper placental microfolds compared to heavier fetuses. In our study, this trend was observed in LLBWP sows through the upregulation of *HBEGF* (*heparin-binding epidermal growth factor*). HBEGF is a molecular mediator of blastocyst implantation, which signals between the endometrium and implanting trophoblast cells to synchronize their corresponding developmental stages. In pigs, *HBEGF* expression by trophoblast cells of the developing placenta appears to regulate extra villous differentiation and provide cytoprotection as a manner of compensation [59,60]. In addition, Stenhouse et al. (2019) [29] demonstrated that it is not only the width and remodeling of the bilayer but also its vascularity that changes to compensate for and rescue the size of the fetus. Specifically, key genes related to placental and embryo development altered their expression during the second phase of angiogenesis [29,30,61,62]. The absence of other genes related to compensatory mechanisms in our study may be explained by the fact that D30 of gestation lies in between the two waves of angiogenesis, and therefore the period of greater compensatory activity had not yet been reached. In contrast to other studies, we did not evaluate extreme low weight conceptuses caused by intrauterine growth retardation, so the mechanisms of compensation were not expected to be as dramatic as in these other cases.

Lastly, another compensatory molecular mechanism we propose is through the activation of transcriptional factors to regulate two different biological processes. Among the four DEGS upregulated transcriptional factors, three of them (*EGR4*, *FOSL1*, *SOX2*, but not *FOXD2*) were related to the regulation of cell proliferation (Figure 3), and may be able to regulate the genes (*CDSN*, *DSG3*, *KLK14*, *KRT17*) involved in cornification and keratinization. Through the TFBS analysis, we predict that all four genes have EGR4 and FOSL1 binding sites at the 5′ non-coding region (Table S5) in order to upregulate the gene expression during the cornification and keratinization processes in the placental tissues of LLBWP sows. On the other hand, regulation through the transcriptional factors of the complex network of genes interacting in placental tissues from LLBWP sows may support physiological compensation of the fetuses as a response to maximize growth and survival.

## Figures and Tables

**Table 1:** Relative expression comparison between LLBWP and HLBWP in D30 placental tissues obtained from the RNA-seq and RT-qPCR studies.

	RNAseq		RT-qPCR	
Genes	log2FoldChange	*p*-Value	log2FoldChange	*p*-Value
	(LLBWP/HLBWP)		(LLBWP/HLBWP)	
*CDSN*	1.62	0.0165551	3.55	0.0057
*HBEGF*	1.48	0.0110607	3.33	0.002
*PDPN*	1.5	0.003	2.14	0.0018

## Data Availability

The data that support the findings of this study are available from the corresponding author upon reasonable request.

## Supplementary Materials

The following supporting information can be downloaded at https://www.mdpi.com/article/10.3390/genes15060703/s1. Table S1: Sequence information for the quantitative real-time RT-PCR validation. Table S2: Clean read quality metrics of all placental samples. Table S3: Gene mapping statistics for all placental samples. Table S4: Full gene annotation. Table S5: Differentially expressed genes in LLBWP placental tissues compared to HLBWP. Table S6: Porcine reference gene list. Table S7: Functional enrichment analysis. Table S8: Transcriptional factor binding site prediction.
